# Person-centred language for describing stratified approaches to TB treatment

**DOI:** 10.5588/ijtldopen.24.0593

**Published:** 2025-01-01

**Authors:** M. Nash, A. Deluca, E. Lessem, N. West, L. McKenna, E.V. McConnell, K. Angami, R. Herrera, A. Makone, G.E. Velásquez, R.E. Chaisson, P.P.J. Phillips

**Affiliations:** ^**1**^ Center for Tuberculosis Research, Department of Medicine, Johns Hopkins University, Baltimore, MD, USA;; ^2^ UCSF Institute for Global Health Sciences (IGHS), University of California, San Francisco, San Francisco, CA, USA;; ^3^ UCSF Center for Tuberculosis, University of California, San Francisco, San Francisco, CA, USA;; ^4^ Treatment Action Group, New York, NY, USA;; ^5^ Access to Rights and Knowledge Foundation, Kohima, India;; ^6^ Universidad Autónoma de Durango, Mexicali Campus, Mexico;; ^7^ Department of Political Science, Stellenbosch University, Cape Town, South Africa;; ^8^ Division of HIV, Infectious Diseases, and Global Medicine, University of California, San Francisco, San Francisco, CA, USA.

**Keywords:** tuberculosis, stigma, stigmatizing language

Dear Editor,

The prevailing ‘one-size-fits-all’ treatment paradigm for TB is changing with the recognition of the potential benefits of stratifying treatment based on the characteristics of the person with TB and disease presentation.^1-4^ This shift is part of a larger movement to make TB care more effective, safe and person-centered. Another key aspect is ensuring the language used in TB care is purposeful, clear and non-stigmatizing.^5,6^ This raises the question of the most appropriate language to describe novel stratified treatment approaches to TB treatment. We therefore conducted a survey to understand the preferred terms used to describe stratified approaches to TB treatment among TB-affected communities, researchers, and providers.

A short, anonymous online English language survey was conducted from May 10 to June 4, 2024. Informed by initial feedback from community partners reviewing a stratified medicine trial protocol, 10 pairs of terms were proposed. Respondents were asked to rate each as ‘preferred’, ‘acceptable’, or ‘unacceptable’ and provide open-ended reasons for their choice. We advertised our survey widely through directed e-mails to TB and HIV research networks’ community advisory bodies, community organizations, and TB civil society listservs. The study was approved by The Johns Hopkins Medicine Institutional Review Board, Baltimore, MD, USA.

In total, 108 individuals completed the survey. Respondents could select multiple identities, but most self-identified as members of civil society (66%) and/or people with lived experience with TB (43%). Healthcare providers (29%), researchers (23%) and TB program staff (21%) were represented. Respondents represented all WHO regions, with over half from the African region (57%), 19% from the South-East Asian Region, 16% from the region of the Americas (excluding the United States and Canada), 15% from the United States and Canada, 7% from Northern/Western/Southern Europe (including the United Kingdom), 3% from Eastern Europe, 2% from the Eastern Mediterranean region, and 1% from the Western Pacific region. All age groups (six categories ranging from 18 to 65+) were represented, with the majority (31%) being between 35 and 44 years old. The most preferred pair of terms was ‘shorter treatment and longer treatment,’ with 46% of respondents indicating these terms as ‘preferred’ (Table). These terms were the top preference across different demographic categories. The second and third most preferred terms also focused on the length of treatment: ‘likely to benefit from shorter treatment and likely to benefit from longer treatment’ and ‘shorter to treat TB and longer to treat TB’. The terms most likely to be unacceptable were ‘less likely to benefit from intensive treatment and more likely to benefit from intensive treatment,’ with 45% of respondents endorsing the pair as ‘unacceptable.’

**Table.** Terms and respondent ratings. Ten terms were proposed to describe stratified treatment options for TB across four categories: treatment length, treatment intensity, participant risk factors and disease complexity. Respondents rated each of the ten proposed terms as ‘unacceptable,’ ‘acceptable or ‘preferred’.

	Unacceptable *n* (%)	Acceptable *n* (%)	Preferred *n* (%)
**Terms about treatment length**			
Shorter/longer treatment	9 (9)	44 (44)	46 (46)
Likely to benefit from shorter/longer treatment	19 (20)	47 (49)	30 (31)
Shorter/longer to treat TB	20 (20)	51 (50)	30 (30)
**Terms about treatment intensity**			
Less/more intensive treatment	25 (26)	54 (56)	17 (18)
Less/more likely to benefit from intensive treatment	43 (45)	41 (43)	12 (12)
**Terms about risk factors**			
Fewer/more risk factors	23 (24)	46 (48)	26 (27)
Higher/lower risk	28 (29)	42 (43)	27 (28)
**Terms about disease complexity**			
Easier/harder to treat TB	29 (28)	54 (52)	21 (20)
Less/more complicated TB	34 (34)	49 (49)	18 (18)
Less/more complex TB	32 (34)	39 (41)	24 (25)

Open-ended responses synthesized overall and by term pairs, provided additional details on respondents’ general preference for the terms focused on treatment length and terms interpreted as simple and understandable:

I am in favor of keeping language simple and direct where possible. So, my preferred term was the shortest one without stigmatizing language.

Reasons for unacceptable terms included the perception that terms are stigmatizing and not person-centered:

I have deprioritized the ‘risk factors’ and ‘lower/higher risk’ as these may impart a sense of there is something about the person or the person’s behaviour that makes them more at risk or risky.

Some respondents described how terms centered on disease complexity may impact engagement and motivation in TB care and treatment:

If I were told I had ‘more complicated TB’ that was ‘harder to treat’, I would feel worse about my prospects than if told I had a form of TB that required longer treatment.

Whereas another highlighted a potential impact on adherence:

Words like ‘risk’, ‘complex’ and ‘complicated’ [dis]empower people and make them question their ability to surmount the challenge of treatment. They put a psychological stumbling block in the patient’s way, and this has huge implications for adherence.

It is important to note that the results were not unanimous: whereas 46% of respondents indicated ‘shorter treatment and longer treatment’ as ‘preferred,’ 9% found those terms ‘unacceptable.’ Conversely, whereas 45% indicated ‘less likely to benefit from intensive treatment and more likely to benefit from intensive treatment’ as ‘unacceptable,’ 12% indicated the terms as ‘preferred.’

We recognize the limitations of our research: we relied on a small, convenience sample and our distribution reached primarily people already engaged in the global TB movement, with internet access, and the survey was only available in English (although three respondents noted they filled out the survey using translated text). Although this was an engaged audience, because the survey was not nested within a clinical trial and many respondents indicated limited experience with clinical trials, this may have been their first time hearing the term ‘stratified medicine’. We hope to conduct preference studies in the future, specifically among trial participants, to enhance our existing research and further refine the language.

The TB community’s pursuit of more purposeful and less stigmatizing language is laudable.^5,7^ Continuing to live up to this goal means that all new TB terms must be selected with intention, ideally in an evidence-based way, with input from TB-affected communities. Previously published descriptions of stratified treatment options for TB have employed terms such as ‘easy-to-treat’ and ‘hard-to-treat’^1^ and ‘low-risk’ and ‘high-risk.’^2^ Our survey findings provide new evidence that people affected by TB and people involved in TB care prefer terms focused on the length of treatment rather than terms focused on the anticipated difficulty of treatment or a person’s risk factors for unfavorable outcomes. Specifically, our survey found that the most preferred terms to describe stratified treatment options were ‘shorter treatment’ and ‘longer treatment.’ These findings will inform the SMART4TB Consortium’s approach to language in our ongoing stratified medicine trials, namely PRISM-TB^8^ and PRISM-TB Kids.^9^ It is worth emphasizing that the importance of language choice in medicine goes beyond individual patient encounters. The use of consistent and optimal language across TB research, advocacy and policy will limit confusion and may increase the acceptability of new treatments. We encourage the broader research and clinical community to consider the language preferences of affected people when writing and communicating about stratified treatment approaches.
